# Using the Knowledge to Action Framework to Describe a Nationwide Implementation of the WHO Surgical Safety Checklist in Cameroon

**DOI:** 10.1213/ANE.0000000000004586

**Published:** 2019-12-16

**Authors:** Michelle C. White, Leonid Daya, Fabo Kwemi Brice Karel, Graham White, Sonia Abid, Aoife Fitzgerald, G. Alain Etoundi Mballa, Nick Sevdalis, Andrew J. M. Leather

**Affiliations:** From the * Centre for Global Health and Health Partnerships, King’s College London, London, United Kingdom; † Department of Anaesthesia, Great Ormond Street Hospital, London, United Kingdom; ‡ Department of Medical Capacity Building, Mercy Ships Africa Bureau, Cotonou, Benin; § Department of Anaesthesia and Intensive Care, Faculty of Medicine and Biomedical Sciences of Yaounde, Yaounde, Cameroon; ‖ Yaounde Emergency Center, Yaounde, Cameroun; ¶ Department of Anaesthesia, Royal Alexandra Hospital, Paisley, United Kingdom; # Department of Medical Capacity Building, Mercy Ships Africa Bureau, Cotonou, Benin; ** Imperial School of Anaesthesia, London, United Kingdom; †† Department of Intensive Care, Oxford University Hospitals, Oxford, United Kingdom; ‡‡ Ministry of Public Health, Cameroon; §§ Faculty of Medicine and Biomedical Sciences of Yaounde, Yaounde, Cameroon; ‖‖ Centre for Implementation Science, King’s College London, London, United Kingdom.

## Abstract

**BACKGROUND:**

Surgical safety has advanced rapidly with evidence of improved patient outcomes through structural and process interventions. However, knowledge of how to apply these interventions successfully and sustainably at scale is often lacking. The 2019 Global Ministerial Patient Safety Summit called for a focus on implementation strategies to maintain momentum in patient safety improvements, especially in low- and middle-income settings. This study uses an implementation framework, knowledge to action, to examine a model of nationwide World Health Organization (WHO) Surgical Safety Checklist implementation in Cameroon. Cameroon is a lower-middle-income country, and based on data from high- and low-income countries, we hypothesized that more than 50% of participants would be using the checklist (penetration) in the correct manner (fidelity) 4 months postintervention.

**METHODS:**

A collaboration of 3 stakeholders (Ministry of Health, academic institution, and nongovernmental organization) used a prospective observational design. Based on knowledge to action, there were 3 phases to the study implementation: problem identification (lack of routine checklist use in Cameroonian hospitals), multifaceted implementation strategy (3-day multidisciplinary training course, coaching, facilitated leadership engagement, and support networks), and outcome evaluation 4 months postintervention. Validated implementation outcomes were assessed. Primary outcomes were checklist use (penetration) and fidelity; secondary outcomes were perioperative teams’ reactions, learning and behavior change; and tertiary outcomes were perioperative teams’ acceptability of the checklist.

**RESULTS:**

Three hundred and fifty-one operating room staff members from 25 hospitals received training. Median time to evaluation was 4.5 months (interquartile range [IQR]: 4.5–5.5, range 3–7); checklist use (penetration) increased from 20% (95% confidence interval [CI], 16–25) to 56% (95% CI, 49–63); fidelity for adherence to 6 basic safety processes was high: verification of patient identification was 91% (95% CI, 87–95); risk assessment for difficult intubation was 79% (95% CI, 73–85): risk assessment for blood loss was 88% (95% CI, 83–93) use of pulse oximetry was 93% (95% CI, 90–97); antibiotic administration was 95% (95% CI, 91–98); surgical counting was 89% (95% CI, 84–93); and fidelity for nontechnical skills measured by the WHO Behaviorally Anchored Rating Scale was 4.5 of 7 (95% CI, 3.5–5.4). Median scores for all secondary outcomes were 10/10, and 7 acceptability measures were consistently more than 70%.

**CONCLUSIONS:**

This study shows that a multifaceted implementation strategy is associated with successful checklist implementation in a lower-middle-income country such as Cameroon, and suggests that a theoretical framework can be used to practically drive nationwide scale-up of checklist use.

**KEY POINTS**

- **Question:** Can the World Health Organization (WHO) Surgical Safety Checklist be implemented nationwide in a lower-middle-income country such as Cameroon, and can a theoretical implementation framework be applied practically to drive scale-up?
- **Findings:** The practical application of the knowledge to action framework using a multidimensional implementation strategy resulted in successful implementation of the WHO Surgical Safety Checklist in Cameroon, which was comparable to other studies in low- (Benin and Madagascar) and high-income countries (United Kingdom).
- **Meaning:** The practical application of implementation research principles offers promise for improved success and sustainability of perioperative improvement interventions in low- and middle-income countries and potentially also in high-income countries.

“Quality should not be the purview of the elite or an aspiration for some distant future; it should be the DNA of all health systems.”^1^ In the past 20 years, much progress has been made in identifying the causes of errors in perioperative care and understanding the relationship between safety culture and patient outcomes. We now know that patient outcomes are not only improved by structural interventions such as increased nurse to patient ratios or intensive care physician involvement in postoperative care but also by interventions that improve health care delivery processes.^2^ Examples include use of checklists, national clinical audits with data feedback, adherence to standardized care pathways, and multidisciplinary team training.^2,3^

However, evidence of how to successfully implement these advances at a national and a global level is lacking in both high- (HICs) and low- and middle-income countries (LMICs).^4,5^ For example, 2 nationwide surgical and intensive care quality improvement interventions in the United Kingdom, Matching Michigan,^6,7^ and the Enhanced Peri-Operative Care for High-risk patients (EPOCH) trial,^8,9^ failed to demonstrate success when scaled up at a national level. The science of perioperative safety has thus moved from an evidence gap to an implementation gap. The 2019 Global Ministerial Patient Safety Summit declared that health care systems must urgently focus on implementation strategies designed to reduce the implementation gap especially in LMICs, if the momentum of the global patient safety movement is to be realized.^10^

The World Health Organization (WHO) Surgical Safety Checklist is a good example of an implementation gap waiting to be addressed in LMICs.^11^ There is overwhelming evidence that the checklist, when properly utilized, improves patient outcomes,^12–14^ yet a recent review highlights increasing evidence of barriers to checklist implementation,^15^ which in turn negatively impacts clinical effectiveness. This is because, as with any clinical intervention, the checklist is only as effective as its implementation. Despite more than a decade of checklist use worldwide, the most effective implementation strategies are poorly understood in HICs and LMICs.

This study uses a well-established implementation framework, knowledge to action (KTA),^16^ to structure a nationwide implementation of the checklist in Cameroon. Cameroon is a LMIC in sub-Saharan Africa with a population of more than 25 million. Implementation frameworks are theoretical models designed to address implementation gaps, such as those described previously. The aim of the study was primarily to evaluate the effectiveness of nationwide checklist implementation in Cameroon, and secondarily to expand our understanding of checklist scale-up by using a theoretical framework (KTA) applied in a clinical context (nationwide checklist implementation)—thereby closing the implementation gap for perioperative safety in LMICs and learning lessons that might have potential for HICs.

## METHODS

Mercy Ships Institutional Review Board (MS-2017-006), King’s College London Research Ethics Service (MR/17/18-399) and the Cameroon Ministry of Health approved the study. All participants voluntarily participated in the study and gave written informed consent. No incentive payments were made. The study is reported in accordance with the STrengthening the Reporting of Observational studies in Epidemiology (STROBE) guidelines.^17^

The study used a collaborative stakeholder approach between the nongovernmental organization Mercy Ships, the Cameroon Ministry of Health, and the academic institution King’s College London, London, United Kingdom. Mercy Ships operates the world’s largest nongovernmental hospital ship, the Africa Mercy. At the invitation of the Head of State, the Africa Mercy typically spends 10 months docked in the main port city of sub-Saharan African countries and provides free surgeries and training.

### KTA Theoretical Framework

**Figure 1.**
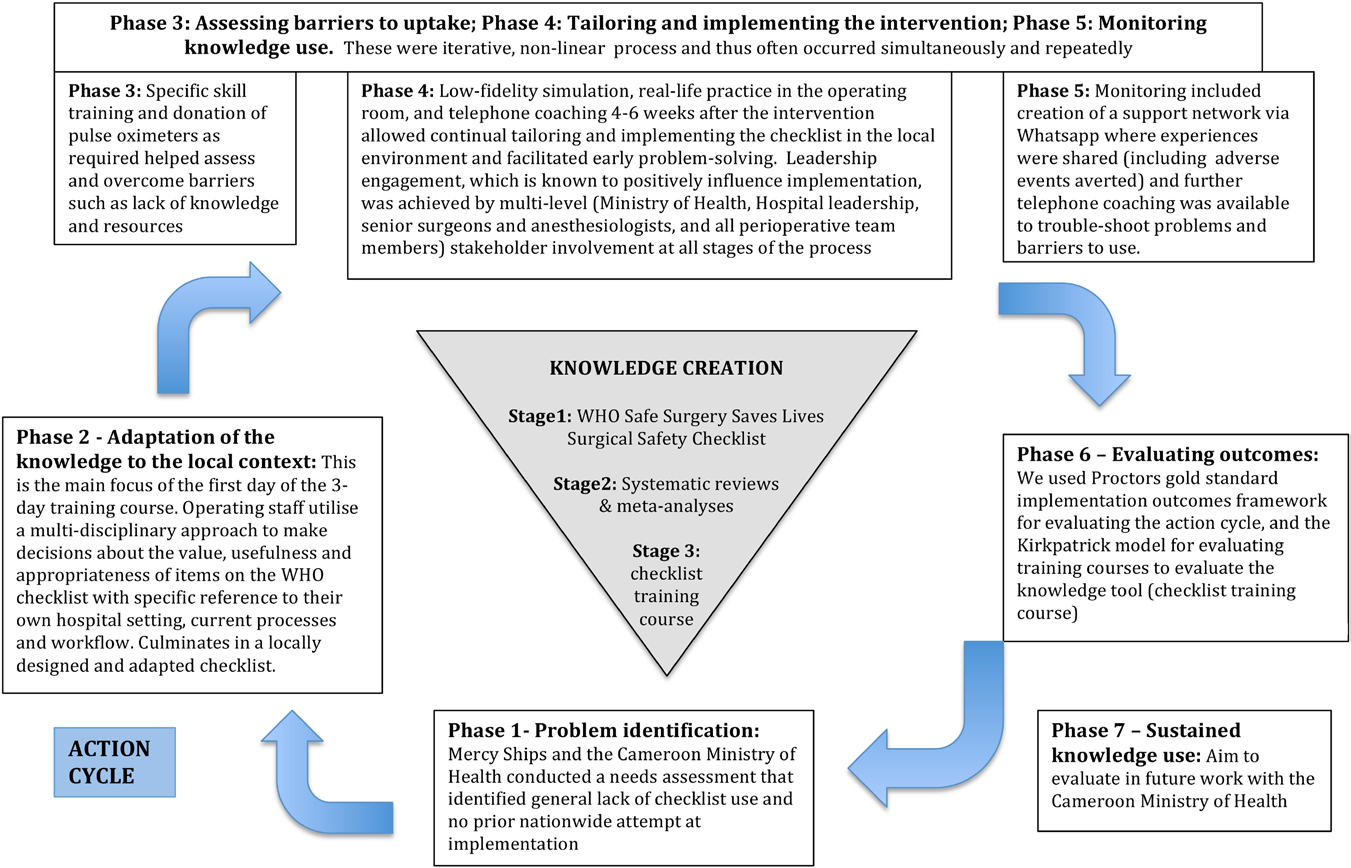
The knowledge to action framework as applied to WHO Surgical Safety Checklist implementation in Cameroon. WHO indicates World Health Organization.

The KTA framework^16^ is a theoretical framework for describing the process of moving clinical evidence into frontline utilization (ie, reducing the implementation gap). Figure 1 depicts how the KTA approach was applied in the study and further details are given in Supplemental Digital Content, Appendix 1, http://links.lww.com/AA/C980.

In brief, the KTA consists of 2 components, knowledge creation and an action cycle.

Knowledge creation aims to create “knowledge tools,” such as practice guidelines or training courses. Knowledge creation is the descriptive process by which knowledge about an intervention passes through several stages: accumulative evidence, aggregation of evidence (eg, via systematic reviews or meta-analyses), and practical synthesis of evidence (eg, practice guidelines, pathways, or training courses). Thus knowledge becomes more distilled, refined, and ultimately more usable to stakeholders.

The action cycle describes a dynamic process of knowledge application, which is deliberately designed to change current ways of doing things, so that the innovative, evidence-based interventions are taken up and used in practice.

### Study Design

We used a prospective observational design consisting of 3 phases:

1. Codesigned problem identification.
2. Codesigned multifaceted checklist implementation.
3. Outcome evaluation at 4 months postintervention.

### Phase 1: Problem Identification (July 2016–July 2017)

In collaboration with the Ministry of Health, Mercy Ships routinely undertakes a needs assessment before codesigning training activities in a country. The needs assessment takes place 12–18 months before the 10-month deployment of Mercy Ships’ hospital ship, and is based on a standard format that consists of hospital surveys; interviews with local surgeons, anesthesiologists, and hospital directors; and discussions with the leaders of the national anesthesia and surgical professional societies, the WHO country director, and the Ministry of Health. In Cameroon, the needs assessment identified that there had been no prior attempts of nationwide WHO checklist implementation and there was a generalized lack of checklist use throughout Cameroon’s main surgical hospitals. Therefore, nationwide implementation of the WHO checklist was agreed upon by the Ministry of Health as a necessary and feasible quality improvement initiative. The precise baseline use of the checklist in each participating hospital was determined in phase 3 of the study.

### Phase 2: Multifaceted Checklist Implementation (August 2017–June 2018)

We aimed to reach all the main government hospitals undertaking surgery across all 10 regions of Cameroon. The Cameroon Ministry of Health selected 34 surgical hospitals to receive a 3-day training course and participate in the study.

We have developed a 3-day multidisciplinary training course (Supplemental Digital Content, Appendix 2, http://links.lww.com/AA/C981) designed for rapid nationwide scale-up of checklist implementation that overcomes known barriers.^18^ The course presents the evidence base for the checklist, encourages teamwork and communication through a multidisciplinary approach to adapting the checklist to the local context; uses low-fidelity simulation to further adapt the checklist; and progresses to real-life supervised practice in the operating room. Thus, there is a dynamic process of locally owned multidisciplinary adaptation of the checklist to each unique hospital context. Component parts of the checklist that may not be familiar, such as the use of pulse oximetry and counting of surgical needles, swabs, and instruments, are taught as part of the course. At the end of the course, pulse oximeters are donated if needed (such that each operating room and recovery area is equipped with a pulse oximeter), and operating rooms are provided with a laminated copy of their own adapted checklist and surgical counting sheet. Hospital directors and senior hospital management (ie, the local leadership figures) are invited to the opening and closing ceremonies of the training to encourage understanding and buy-in.

The Cameroon Ministry of Health contacted each hospital to inform them of the overall nationwide checklist implementation plan and to request attendance of all surgical staff at the trainings. Because most surgical hospitals do not run the operating rooms to full capacity, it was agreed to suspend elective operating during the training course, as it was deemed that sufficient capacity existed to schedule the work later. Emergency work continued uninterrupted, which meant the on-call staff missed aspects of the training if attending an emergency. However, 8 hospitals in the Yaounde region did not have the capacity to suspend elective work for the duration of the course. These hospitals were excluded, leaving 26 participating hospitals.

The 3-day training course was taught in each hospital by a 5-person training team consisting of 2 Cameroonian doctors, 2 British doctors, and a British operating room nurse.

During the course, we identified local “checklist champions” who were enthusiastic about the checklist and/or people of influence in the operating room. These individuals received telephone coaching for 2–4 weeks postcourse to provide support and troubleshoot any problems. All “checklist champions” received at least 1 telephone call from the training team, with further calls arranged as needed. All participants were invited to join a WhatsApp support group established and moderated by the 2 Cameroonian doctors from the training team. The group aimed to encourage sharing of implementation experiences and problem-solving.

### Phase 3: Outcome Evaluation (March 2018–June 2018)

Checklist implementation was evaluated using 2 gold-standard validated frameworks: the Kirkpatrick framework for the evaluation of complex training interventions^19^ and Proctor’s implementation outcomes framework^20^ (Table 1).

**Table 1.** Outcome Evaluation Frameworks: Definitions and Measurement Tools Used in Current Study

Framework	Definition	Measurement Tool Used
Kirkpatrick framework for evaluating complex training interventions		
Level 1: reaction	The degree to which participants find the training favorable, engaging and relevant to their jobs	Questionnaire 2
Level 2: learning	The degree to which participants acquire the intended knowledge, skills, attitude, confidence, and commitment based on their participation in the training	Questionnaire 2
Level 3: behavior	The degree to which participants apply what they learned during training when they are back on the job	Questionnaire 1
Proctor implementation outcomes framework		
Acceptability	Perception among stakeholders that the intervention is agreeable	Questionnaire 3
Adoption	Willingness to start using the intervention	Questionnaire 2^a^
Appropriateness	Perception among stakeholders of the fit and relevance of the intervention to the local context	Questionnaire 2^a^
Feasibility	Extent to which an intervention can be successfully performed	Questionnaire 2^a^
Fidelity	Degree to which the intervention is implemented as originally intended	Questionnaire 1 WHOBARS
Penetration	Integration of an intervention within a service system	Questionnaire 1

Abbreviation: WHOBARS, World Health Organization Behaviorally Anchored Rating Scale.

^a^Adoption, appropriateness, and feasibility were inferred from Questionnaire 2.

Based on previous checklist implementations in LMICs and HICs,^21–23^ we hypothesized that more than 50% of participants would be using the checklist at 4 months (penetration) and in the correct manner (fidelity).

Data were collected at 3 time points: (1) before the course; (2) immediately after the course; (3) 4 months after the course.

Primary outcomes were checklist penetration (overall use) and fidelity (how well used) at 4 months postintervention. Secondary outcomes were operating room staff’s reactions, learning, and behavior change associated with the training course; and tertiary outcome was participating teams’ acceptability of the checklist at 4 months postintervention.

Outcomes were assessed as follows:

Primary outcomes (checklist penetration and fidelity): Questionnaire 1 is a self-reported validated questionnaire designed to measure penetration and fidelity.^22,23^ The questionnaire uses a 5-point Likert scale response format to measure overall checklist use (penetration) and the use of 6 key safety steps^12^ (fidelity): verification of patient identity and site of intervention, assessment of difficult intubation risk, evaluation of the risk of major blood loss, use of a pulse oximeter, timely administration of antibiotics, and surgical counts. The questionnaire also evaluates participant’s behavior change at 4 months. Fidelity was further assessed using the validated World Health Organization Behaviorally Anchored Rating Scale (WHOBARS) tool in vivo.^24^ The designers of the WHOBARS tool had trained Mercy Ships staff in the use of the WHOBARS tool in 2017, and since then, Mercy Ships have used WHOBARS to evaluate checklist use in various LMICs. WHOBARS assesses nontechnical skills during checklist administration. For each part of the checklist (sign in, time out, sign out), WHOBARS evaluates 5 domains on a scale from 1 to 7: setting the stage, team engagement, communication activation, communication of problem anticipation, and communication of process completion. Scores are combined and then averaged to give an overall WHOBARS score (range 1–7), with higher scores indicating superior nontechnical skills, conducive to high-fidelity checklist application.

Secondary outcomes (reactions, learning, and behavior change): Questionnaire 2 is a self-reported questionnaire designed to measure participant’s reaction and learning (Kirkpatrick level 1 and 2) associated with the training course using a 1–10 visual analog scale (1 = not at all, 10 = very much) in response to the following questions:

1. Did you enjoy the course? (reaction)
2. Did you find the course helpful? (reaction)
3. Do you feel more confident in the skills that were taught? (learning)
4. Do you think the training will help improve your practice? (learning)
5. Will you share the information you learned with others, for example, your students or your colleagues? (learning)

Tertiary outcome (acceptability 4 months postintervention): Questionnaire 3 is a self-reported, previously reported questionnaire^22^ that uses 5-point Likert scale to measure acceptability of the checklist at 4 months over 7 domains:

1. The checklist improves the communication in the operating room.
2. The checklist improves the organization in the operating room.
3. The checklist helps with infection prevention and control.
4. The checklist improves patient care.
5. The checklist improves my personal satisfaction in my job.
6. The checklist reduces the stress I feel carrying out my role.
7. The checklist improves the culture of patient safety in the hospital.

Questionnaire 1 was administered immediately before the course and at 4 months after, Questionnaire 2 immediately after the course, and Questionnaire 3 at 4 months. WHOBARS assessments were made during site visits to each hospital at 4 months. If surgery occurred during the site visit, we made direct observations of checklist use in real-time in the operating room. If no surgery occurred during the site visit, we used simulation where participants adopted their usual professional role. The training team made the assessments immediately before and after the course, and a Mercy Ships evaluation team (which included the Cameroonian doctors from the training team) made the assessments at 4 months.

### Statistical Analysis

Descriptive statistics are reported for all outcome measures. The primary outcome (checklist penetration) Likert scores were dichotomized into “always/often” and “sometimes/rarely/never.” These binary outcomes were compared before and after the intervention using a McNemar test (with Yates correction), with *P* < .01 considered significant.

## RESULTS

The 26 hospitals were deidentified as hospitals A–Z. No hospitals, except hospital P, were routinely using the checklist as a standard of care before the study, therefore hospital P was excluded from analysis, leaving 425 participants from 25 hospitals that received checklist training. Three hundred fifty-one of 425 staff trained consented to data collection immediately postcourse. The sample comprised 57 surgeons, 53 anesthesia providers, 136 nurses, 41 other (biomedical technicians, sterile processing technicians, medical or nursing students), and 64 had no title recorded. In Cameroon, anesthesia is provided by both physician and nonphysician anesthetists who, for the purposes of the study, were all counted as “anesthetists.”

Median time from initial training to evaluation was 4.5 months (interquartile range [IQR] 4.5–5.5, range 3–7). Site visits at 4 months were made to 18/25 hospitals. Seven were outside of the security zone due to civil disturbances at the time of the study and received telephone follow-up only. As we were unable to gain written consent, administer the questionnaires or WHOBARS assessments, these hospitals were excluded from the final analysis. One hundred eighty-three staff consented to data collection at the 4-month evaluation (23 surgeons, 42 anesthetists, 82 nurses, 17 other, 19 no title recorded), giving a follow-up rate of 52% (183/351).

### Primary Outcomes: WHO Checklist Penetration and Fidelity

Reported use of checklist and team brief (always/often) increased from 20% (95% confidence interval [CI], 16–25) to 56% (95% CI, 49–63) (*P* < .001) and 15% (95% CI, 11–19) to 60% (95% CI, 52–67) (*P* < .001), respectively. Fidelity of checklist administration at 4 months was good, as shown by high use (always/often) of the 6 key safety processes: verification of patient identification was 91% (95% CI, 87–95); risk assessment for difficult intubation was 79% (95% CI, 73–85): risk assessment for blood loss was 88% (95% CI, 83–93) use of pulse oximetry was 93% (95% CI, 90–97); antibiotic use was 95% (95% CI, 91–98); and surgical counting was 89% (95% CI, 84–93). Details are shown in Table 2.

**Table 2.** Summary of Use of WHO Surgical Safety Checklist, Team Brief, Basic Safety Processes Before and After Training

	Always (%)	Often (%)	Sometimes (%)	Rarely (%)	Never (%)	NR (%)
Surgical Safety Checklist used	34 (10)^a^	32 (10)^a^	46 (14)^a^	22 (7)^a^	180 (56)^a^	10 (3)^a^
	55 (30)^b^	48 (26)^b^	59 (32)^b^	11 (6)^b^	6 (3)^b^	4 (2)^b^
Team brief completed	26 (8)^a^	23 (7)^a^	53 (16)^a^	53 (16)^a^	86 (27)^a^	83 (26)^a^
	59 (32)^b^	75 (41)^b^	23 (13)^b^	16 (9)^b^	7 (4)^b^	3 (2)^b^
Verification of patient identity	211 (65)^a^	57 (18)^a^	31 (10)^a^	11 (3)^a^	11 (3)^a^	3 (1)^a^
	142 (78)^b^	24 (13)^b^	7 (4)^b^	1 (1)^b^	7 (4)^b^	2 (1)^b^
Risk of difficult airway assessed	104 (32)^a^	56 (17)^a^	68 (21)^a^	38 (12)^a^	34 (10)^a^	24 (7)^a^
	107 (58)^b^	37 (20)^b^	11 (6)^b^	10 (5)^b^	7 (4)^b^	11 (6)^b^
Risk of blood loss assessed	134 (41)^a^	86 (27)^a^	63 (19)^a^	21 (6)^a^	15 (5)^a^	5 (2)^a^
	125 (68)^b^	36 (20)^b^	7 (4)^b^	10 (5)^b^	1 (1)^b^	4 (2)^b^
Use of pulse oximetry	177 (55)^a^	62 (19)^a^	36 (11)^a^	13 (4)^a^	31 (10)^a^	5 (2)^a^
	153 (84)^b^	18 (10)^b^	5 (3)^b^	2 (1)^b^	0 (0)^b^	5 (3)^b^
Prophylactic antibiotic administration	117 (36)^a^	72 (22)^a^	58 (18)^a^	35 (11)^a^	33 (10)^a^	9 (3)^a^
	141 (77)^b^	32 (17)^b^	5 (3)^b^	1 (1)^b^	1 (1)^b^	3 (2)^b^
Surgical count completed	132 (41)^a^	51 (16)^a^	52 (16)^a^	35 (11)^a^	48 (15)^a^	6 (2)^a^
	102 (56)^b^	60 (33)^b^	13 (7)^b^	4 (2)^b^	3 (2)^b^	1 (1)^b^

Values given are number and percentage.

Abbreviations: NR, not recorded; WHO, World Health Organization.

^a^Before training.

^b^Four months after training.

**Table 3.** Mean Hospital WHOBARS Scores

Hospital	WHOBARS Sign In	WHOBARS Time Out	WHOBARS Sign Out	Overall WHOBARS Score
A	5.1	5.6	5.7	5.5
B	3.0	5.7	2.9	3.9
C	4.5	3.7	3.0	3.7
D	^a^	^a^	^a^	^a^
E	6.1	4.8	^b^	5.5
F	4.8	5.9	2.5	4.4
G	…	…	…	…
H	^c^	^c^	^c^	^c^
I	2.4	2.3	2.3	2.3
J	2.6	4.0	1.6	2.7
K	5.4	5.1	5.6	5.4
L	6.9	6.5	6.9	6.7
M	^a^	^a^	^a^	^a^
N	6.1	6.1	6.4	6.2
O	6.2	6.1	6.4	6.2
P	^d^	^d^	^d^	^d^
Q	7.0	7.0	7.0	7.0
R	5.9	5.1	4.9	5.3
S	5.5	4.9	4.7	5.0
T	^a^	^a^	^a^	^a^
U	^a^	^a^	^a^	^a^
V	^a^	^a^	^a^	^a^
W	^a^	^a^	^a^	^a^
X	3.4	4.6	1.5	3.2
Y	^a^	^a^	^a^	^a^
Z	^a^	^a^	^a^	^a^

WHOBARS scale 1–7.

Abbreviation: WHOBARS, World Health Organization Behaviourally Anchored Rating Scale.

^a^Not evaluated as no site visit at 4 mo.

^b^Not seen as team left before sign out completion.

^c^Not evaluated due to unsupportive surgeon who refused to use the checklist.

^d^Hospital P was excluded from final analysis as was already using the checklist before the intervention.

Fidelity of nontechnical skills during checklist use was moderate, as shown by median WHOBARS of 4.5 of 7 (95% CI, 3.5–5.4). Individual WHOBARS scores are shown in Table 3.

### Secondary Outcomes: Reactions, Learning, and Behavior Change Immediately Postintervention

Evaluation of the training course as measured by participant’s reaction and learning was very good. In response to the 5 questions (listed earlier), median visual analog scores were all 10 (IQR 9–10 for all, and range 5–10, 4–10, 3–10, 3–10, and 5–10 for questions 1–5, respectively).

### Tertiary Outcome: WHO Checklist Acceptability 4 Months Postintervention

**Figure 2.**
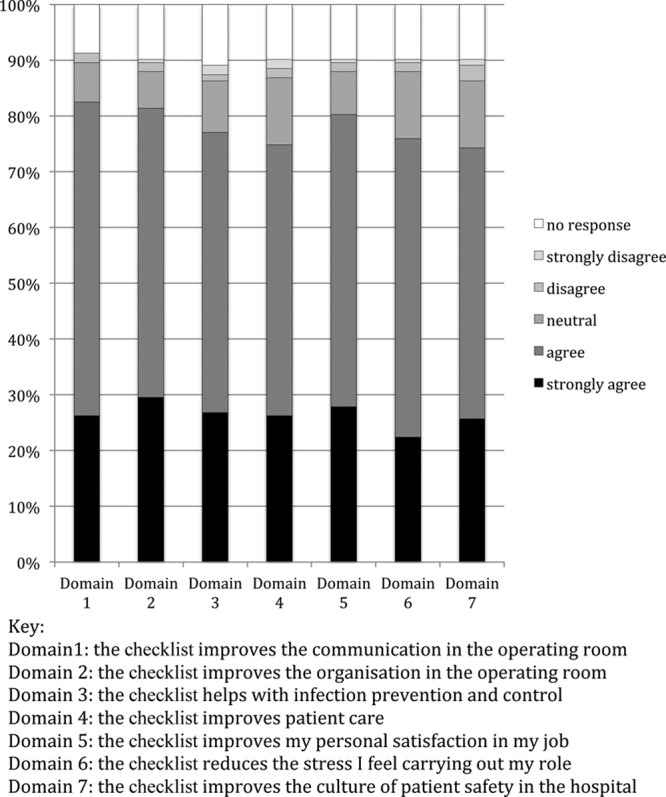
Summary of staff perceptions of the acceptability and benefits of using the WHO Surgical Safety Checklist. WHO indicates World Health Organization.

Participating perioperative teams’ acceptability of the checklist and benefits to staff and patients at 4 months was high; as shown, all variables scoring more than 70%. See Figure 2.

## DISCUSSION

This study reports checklist penetration of 56% postintervention. Fidelity of the 6 basic safety processes was at least 79%, and nontechnical skills were 4.5 of 7. This means that when the checklist is used, it is used properly. Staff generally found using the checklist as acceptable and desirable, reporting benefits to both themselves (improved job satisfaction and reduced stress at work) and their patients (improved infection control and patient safety practices). These results are comparable to other LMIC and HIC studies where checklist use ranges from 30% to 100%.^21–23^

Based on results from this study and studies from Madagascar and Benin,^22,23^ we believe that 2 main reasons determined the success of our approach: the design of the training course and the approach to training. The course was designed specifically to overcome known barriers to implementation,^18^ which include skepticism regarding the evidence base, concerns over time and efficiency, the need for workflow adjustments, lack of psychological ownership, lack of sufficient training and resources, lack of surgeons and executive leadership commitment, and poor communication and teamwork.^25–29^ The approach to checklist training was more than just delivering a training course. We utilized a multifaceted implementation strategy comprising coaching, facilitated leadership engagement at the local hospital and Ministry of Health level, and creation of a WhatsApp support network. In essence, our strategy focused on 4 key areas: overcoming material challenges, improvement of technical skills, multidisciplinary teamwork, and attention to leadership and sustainability.^18^ We suggest these 4 areas may be important for successful scale-up, not just of the checklist but also other perioperative quality improvement initiatives.

Further, we take the view that this study may carry implications for perioperative improvement programs in HICs. Published evidence suggests that both HICs and LMICs may benefit from similar approaches to perioperative safety improvement because the origins and solutions are rooted in 4 common domains: human factors, resources, culture, and behavior.^30,31^ This means that lessons learned in LMICs may be applied to HICs and vice versa, creating mutual learning. The checklist is, in reality, an intervention that requires behavioral change and reinforcement to be applied coherently, and we think we see similar patterns in the required behaviors in our study as others have found in HICs. Implementation research is a form of health policy and systems research that uses theoretical frameworks to study all 4 domains, and thereby support the scale-up of quality improvement interventions and their subsequent integration into health systems at the national level.^32^ Two large trials aiming to scale-up quality improvement interventions in the United Kingdom are thought to have been unsuccessful due to a failure of implementation. The EPOCH trial^8^ attempted to implement an evidence-based multimodal care pathway for patients undergoing emergency laparotomy across 93 UK hospitals, but was unable to demonstrate improvement in survival or length of hospital stay. Matching Michigan^6^ aimed to reduce central venous catheter infections across 223 UK intensive care units but was unable to demonstrate benefit. These negative outcomes were thought to be due to challenges of implementation such as under-estimating the importance of the local context and the social aspects of change.^7,9^ Both of these examples used an intervention with a strong evidence base, but the failure to effectively implement the intervention, at scale, highlights the implementation gap facing clinicians in HICs. We propose that as HIC health care professionals, we need a better understanding of implementation research to scale-up locally designed quality-improvement interventions to bring greater equity to health care delivery in our own countries^4,15^; and as a global community, we must begin to move beyond the plethora of publications continuing to define the problems of the lack of access to quality perioperative care to finding real-life solutions.

Our study used the implementation research framework (KTA) to demonstrate how a theoretical approach can be applied to a pragmatic implementation. The importance of implementation strategies for national scale-up of the checklist has been highlighted in the literature. Shortly after the checklist was launched, the WHO unsuccessfully attempted nationwide checklist scale-up in 15 African countries.^33^ Leading surgeons and anesthesiologists from each country were invited to 2 workshops that aimed to develop plans for checklist implementation and nationwide rollout. However, a year later, implementation had succeeded in only 10 hospitals across the 15 countries, and no nationwide scale-up had been attempted. Current evidence of successful checklist implementation in LMICs is limited to small or single-center studies with few large-scale initiatives reported.^22,23,34^ We interpret this evidence as showing that more well-designed and theory-informed implementation studies are needed so that the evidence base on successful and feasible implementation strategies for scale-up can be developed further. This is what the current study offers.

Fifteen years ago, the WHO Ministerial Summit on Health Research^35^ called for more use of implementation research in health systems strengthening, but a recent systematic review showed that little progress has been made.^36^ Not surprisingly, therefore, the recent 2019 Global Ministerial Patient Safety Summit called for health systems to focus on implementation strategies to capitalize on the momentum of the global patient safety movement.^10^ Yet in the surgical and anesthesia literature in LMICs, there are few reports describing implementation strategies for perioperative quality improvement or policy programs at national level. This study adds to the literature by providing a pragmatic example of using a theoretical implementation framework (KTA) and translating this into the clinical context on a national scale.

This study has several important limitations. We used previously validated but self-reported responses. The relationship between self-reported change and actual behavior change and process compliance was not measured. Self-reporting is subject to recall bias, and under- or overreporting actual change: overreporting to please the interviewers (social desirability bias), or under-reporting to obtain more training. Direct observations of checklist use with WHOBARS overcame self-responder bias but were limited due to time and refusal to consent (1 hospital), so were not completed in all sites. WHOBARS data were collected after observing only 1 or 2 cases whereas a sample size of 9 is recommended to show a difference between hospitals.^24^ However, our aim was not to compare hospitals but use WHOBARS to assess the nontechnical aspects of checklist use and make an objective assessment in addition to the self-reported assessments. The study lacked control sites, so we cannot comment on causality. The lack of control sites could be addressed in future studies using a step wedge design. We made no assessment of clinical outcomes, but because the checklist is already well evidenced, the research question is no longer, “does the checklist work?” but rather “how can we make the checklist work in real life and at scale?” We have attempted to begin to answer this question but further studies are needed to facilitate our understanding of improved implementation of the checklist. A direction for future implementation research on the checklist would be to use different implementation frameworks, which have different foci, such as cultural or behavioral change. It may be that different hospital cultures respond better to different strategies. The evaluation team was not independent of the training team, but having an evaluator who is known to participants reduces responder bias.^37,38^ This study involved both a behavioral research component (surveys and WHOBARS) and an implementation framework (KTA). We did not aim to separate the different components of the study; the measures required by the KTA framework were provided through the use of the survey and WHOBARS behavioral assessments, so we cannot comment on what the study findings would have been had different assessments been performed, nor are we able to comment on the successes/deficiencies of the KTA framework compared with other theoretical frameworks. Our study had a follow-up rate of only 52%, considerably lower than at the initial training. We think this is because the hospital director requested attendance at the initial training and postponed elective operating to facilitate this. However, participation in the follow-up was voluntary, and elective surgeries were not postponed. The Ministry of Health had stipulated that it was justified to postpone elective surgery for training purposes but not for research.

This study used a well-established implementation framework (KTA) to present a pragmatic, real-world strategy for successful nationwide scale-up of the checklist in Cameroon. Applying implementation strategies offers promise for improved success and sustainability of perioperative improvement interventions in LMICs and potentially also in HICs.

## ACKNOWLEDGMENTS

The authors thank Ali Herbert, Sophie Corset, Kirsten Randall, and Eiling Wu for help with logistics and data collection.

## DISCLOSURES

**Name:** Michelle C. White, MB ChB.

**Contribution:** This author conceived the original idea; helped with study design; helped with data acquisition, interpretation, and analysis; reviewed the literature; wrote the first draft of the manuscript; and approved the final manuscript.

**Conflicts of Interest:** None.

**Name:** Leonid Daya, MD.

**Contribution:** This author helped acquire the data, and critically appraised and approved the final manuscript.

**Conflicts of Interest:** None.

**Name:** Fabo Kwemi Brice Karel, MD.

**Contribution:** This author helped acquire the data, and critically appraised and approved the final manuscript.

**Conflicts of Interest:** None.

**Name:** Graham White, MB ChB.

**Contribution:** This author helped acquire the data, and critically appraised and approved the final manuscript.

**Conflicts of Interest:** None.

**Name:** Sonia Abid, MB BS.

**Contribution:** This author helped acquire the data, and critically appraised and approved the final manuscript.

**Conflicts of Interest:** None.

**Name:** Aoife Fitzgerald, MB BS.

**Contribution:** This author helped acquire the data, and critically appraised and approved the final manuscript.

**Conflicts of Interest:** None.

**Name:** G. Alain Etoundi Mballa, MD.

**Contribution:** This author helped acquire the data, and critically appraised and approved the final manuscript.

**Conflicts of Interest:** None.

**Name:** Nick Sevdalis, PhD.

**Contribution:** This author helped with study design; data interpretation and analysis; and critically appraised and approved the final manuscript.

**Conflicts of Interest:** N. Sevdalis is the director of the London Safety and Training Solutions Ltd, which offers training in patient safety, implementation solutions, and human factors to healthcare organizations.

**Name:** Andrew J. M. Leather, MS.

**Contribution:** This author helped with study design; data interpretation and analysis; and critically appraised and approved the final manuscript.

**Conflicts of Interest:** None.

**This manuscript was handled by:** Angela Enright, MB, FRCPC.

## Supplementary Material


