# Supplementary material for: Using the Knowledge to Action Framework to Describe a Nationwide Implementation of the WHO Surgical Safety Checklist in Cameroon
Source: Anesth Analg. 2019 Dec 16;130(5):1425–34. doi: 10.1213/ANE.0000000000004586 (PMC7147425; doi:10.1213/ANE.0000000000004586)
Supplement: Supplementary file 1 [file ane-130-1425-s001.docx]

**Appendix 1: Knowledge to Action framework as applied to checklist implementation**

*Knowledge creation* aims to create knowledge tools or products (for example, practice guidelines). During knowledge creation, knowledge about an intervention (here, the WHO checklist) passes through 3 stages becoming more distilled and refined and thus more usable to stakeholders:

1. *Stage 1: Knowledge Inquiry*: For the checklist this is represented by the 2008 WHO Safe Surgery Saves Lives document in which the checklist was first published.^1^ This was based on the cumulative evidence of checklists used in healthcare and other industries and a global trial conducted by Haynes et al that was subsequently published in 2009.^2^
2. *Stage 2: Knowledge Synthesis*: This represents the aggregation of existing knowledge. For the checklist there are a multitude of systematic reviews and meta-analyses to prove it works.^3-6^
3. *Stage 3: Knowledge Tools and Products*: In our study the knowledge tool is the 3-day training course, designed to facilitate implementation of the checklist.

*Action cycle*: this part describes a dynamic process of knowledge application deliberately designed to change current ways of doing things, such that the innovative evidenced interventions are taken up and used in practice. The action cycle describes the implementation process and consists of seven steps – as follows:

1. Identify the problem
2. Adapt knowledge to the local context
3. Assess barriers to knowledge use
4. Select, tailor, implement the knowledge (intervention)
5. Monitor knowledge use
6. Evaluate outcomes
7. Sustain knowledge use

**References**

1. World Health Organisation Safe Surgery Saves Lives. Guidelines for Safe Surgery, Geneva, WHO Press, 2009.

2. Haynes AB, Weiser TG, Berry WR*, et al.* A surgical safety checklist to reduce morbidity and mortality in a global population N Engl J Med 2009: 360; 491-499

3. Abbott TEF, Ahmad T, Phull MK*, et al.* The surgical safety checklist and patient outcomes after surgery: a prospective observational cohort study, systematic review and meta-analysis British Journal of Anaesthesia: 120; 146-155

4. Borchard A, Schwappach DL, Barbir A*, et al.* A systematic review of the effectiveness, compliance, and critical factors for implementation of safety checklists in surgery Annals of surgery 2012: 256; 925-933

5. Bergs J, Hellings J, Cleemput I*, et al.* Systematic review and meta-analysis of the effect of the World Health Organization surgical safety checklist on postoperative complications Br J Surg 2014: 101; 150-158

6. Treadwell JR, Lucas S, Tsou AY Surgical checklists: a systematic review of impacts and implementation BMJ Qual Saf 2014: 23; 299-318
