# Supplementary material for: Using the Knowledge to Action Framework to Describe a Nationwide Implementation of the WHO Surgical Safety Checklist in Cameroon
Source: Anesth Analg. 2019 Dec 16;130(5):1425–34. doi: 10.1213/ANE.0000000000004586 (PMC7147425; doi:10.1213/ANE.0000000000004586)
Supplement: Supplementary file 2 [file ane-130-1425-s002.docx]

**Appendix 2: Summary of the WHO Surgical Safety Checklist course.**

| *Day 1* | Morning:   - Welcome to the course by the training team and the Hospital Director. Baseline data collection. Introduction to patient safety and evidence of the benefit of using the checklist. - Adaptation of the checklist for the local hospital environment. Divided into three multi-disciplinary groups and each given a copy of the original WHO SSC. Each group given one part of the checklist (sign in, time out or sign out) and asked to adapt that part to their own environment. Then the groups rotate so that each group has a chance to adapt each part of the checklist and build on what the previous group adapted. At the end all groups feedback and further adaptations are agreed. - Low-fidelity simulation of using the adapted hospital checklist in a classroom environment using local materials such as patient charts. Further adaptations are made as necessary and suggested by the participants. - Participants lead adaptation process, while instructors facilitate or coach where necessary to maintain fidelity to the original essence of the checklist.   Afternoon: (or may be postponed until Day 2 if morning session not completed):  Divide into two groups to teach key components of the checklist:   1. nurses and surgeons:    1. Counting of compresses and instruments training 2. Physician and non-physician anesthetists:    1. use of a pulse oximetry and management of hypoxia |
| --- | --- |
| *Day 2* | - Low fidelity simulation of using the adapted hospital checklist in the operating room. Simulations become increasingly complex, for example: emergency surgery, difficult intubation, major hemorrhage, lost instruments or sponges. Simulations are designed to address the 6 key safety steps* that the checklist is designed to unsure happen correctly for every patient. Iterative adaptations of the checklist continue as required. - checklist is discussed and further adaptations are made as necessary - Additional counting or pulse oximeter /hypoxia training as necessary - Real-life use of the checklist in the OR with patients and any further iterative adaptations. |
| *Day 3 (optional)* | - Further use of the checklist in the operating room either in real-life situations - Final discussions with, and encouragement for, the operating room teams, including readiness for change, and how to overcome anticipated challenges in using the checklist - Participant feedback - Formal ceremony and handing over of donated equipment and certificates. |

*6 key safety steps: verification of patient identity and site of intervention, assessment of difficult intubation risk, evaluation of the risk of major blood loss, use of a pulse oximeter, timely administration of antibiotics, and surgical counting swabs
